# Nobiletin Attenuates Pathological Cardiac Remodeling after Myocardial Infarction via Activating PPAR*γ* and PGC1*α*

**DOI:** 10.1155/2021/9947656

**Published:** 2021-08-06

**Authors:** Yufei Zhou, Ting Yin, Mengsha Shi, Mengli Chen, Xiaodong Wu, Kai Wang, Iokfai Cheang, Yanxiu Li, Hongcai Shang, Haifeng Zhang, Xinli Li

**Affiliations:** ^1^Department of Cardiology, The First Affiliated Hospital of Nanjing Medical University, Nanjing, Jiangsu, China; ^2^Department of Cardiology, The First Affiliated Hospital of Zhejiang University, Hangzhou, Zhejiang, China; ^3^Department of Critical Care Medicine, The First Affiliated Hospital of Nanjing Medical University, Nanjing, Jiangsu, China; ^4^Key Laboratory of Chinese Internal Medicine of Ministry of Education, Dongzhimen Hospital, Beijing University of Chinese Medicine, Beijing, China; ^5^Department of Cardiology, The Affiliated Suzhou Hospital of Nanjing Medical University, Suzhou, Jiangsu, China

## Abstract

**Materials and Methods:**

C57BL/6 mice were treated with coronary artery ligation to generate an MI model, followed by treatment for 3 weeks with NOB (50 mg/kg/d) or vehicle (50 mg/kg/d), with or without the peroxisome proliferator-activated receptor gamma (PPAR*γ*) inhibitor T0070907 (1 mg/kg/d). Cardiac function (echocardiography, survival rate, Evans blue, and triphenyl tetrazolium chloride staining), fibrosis (Masson's trichrome staining, quantitative real-time polymerase chain reaction (qRT-PCR), and western blot (WB)), hypertrophy (haematoxylin-eosin staining, wheat germ agglutinin staining, and qRT-PCR), and apoptosis (WB and terminal deoxynucleotidyl transferase dUTP nick-end labelling (TUNEL) staining) were evaluated. Hypoxia-induced apoptosis (TUNEL, WB) and phenylephrine- (PE-) induced pathological hypertrophy (immunofluorescence staining, qRT-PCR) models were established in primary neonatal rat ventricular myocytes (NRVMs). The effects of NOB with or without T0070907 were examined for the expression of PPAR*γ* and PPAR*γ* coactivator 1*α* (PGC1*α*) by WB in mice and NRVMs. The potential downstream effectors of PPAR*γ* were further analyzed by WB in mice.

**Results:**

Following MI in mice, NOB intervention enhanced cardiac function across three predominant dimensions of pathological cardiac remodeling, which reflected in decreasing cardiac fibrosis, apoptosis, and hypertrophy decompensation. NOB intervention also alleviated apoptosis and hypertrophy in NRVMs. NOB intervention upregulated PPAR*γ* and PGC1*α in vivo* and *in vitro*. Furthermore, the PPAR*γ* inhibitor abolished the protective effects of NOB against pathological cardiac remodeling during the progression from MI to CHF. The potential downstream effectors of PPAR*γ* were nuclear factor erythroid 2-related factor 2 (Nrf-2) and heme oxygenase 1 (HO-1).

**Conclusions:**

Our findings suggested that NOB alleviates pathological cardiac remodeling after MI via PPAR*γ* and PGC1*α* upregulation.

## 1. Introduction

Chronic heart failure (CHF) is associated with high morbidity and mortality worldwide [1]. Myocardial infarction (MI) is the most prominent cause of CHF [2]. Although artery reperfusion via percutaneous coronary intervention (PCI) can significantly decrease the acute mortality rate, adverse cardiac events often recur, and survivors are susceptible to pathological cardiac remodeling [3].

Pathological cardiac remodeling following MI represents a pivotal mechanism that underlies CHF development and involves three predominant pathways [4]. The cell loss pathway is associated with cardiomyocyte necrosis and apoptosis, the decompensated cardiac hypertrophy pathway is induced by abnormal oxidative stress and energy metabolism, and the fibrosis pathway is manifested by the activation of myofibroblasts and monocyte infiltration [5]. The exposure of cardiomyocytes to hypoxic and ischemic conditions after MI disrupts the Ca^2+^ stability necessary to regulate systolic and diastolic strength, causing mitochondrial damage. Due to the limited regenerative abilities of cardiomyocytes, cardiac infarcts often result in the development of scar tissue, which can contribute to pathological cardiac remodeling [6, 7]. Although the mechanisms underlying cardiac remodeling are well understood, few therapeutic targets exist to alleviate the pathological cardiac remodeling that occurs after MI; thus, the identification of novel targets remains an urgent goal.

**Figure 1:**
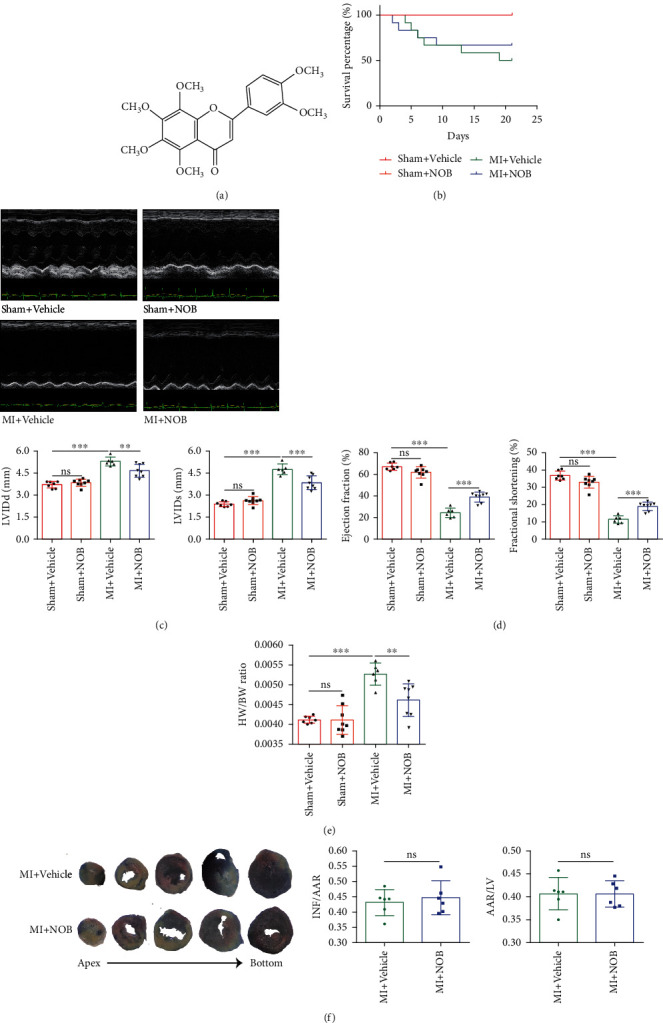
NOB protects against CHF after MI. (a) The chemical structure of NOB. (b) The survival curve showed that NOB intervention improved the survival rate of mice after MI. The numbers of mice for the sham+vehicle, sham+NOB, MI+vehicle, and MI+NOB were 7, 8, 12, and 12 at the start of the experiment and after 3 weeks, and the numbers were 7, 8, 6, and 8, respectively. Six mice were sacrificed in the MI+vehicle group, and four were sacrificed in the MI+NOB group. The survival rates for each group were 100%, 100%, 50%, and 66.7%, respectively. (c) Representative echocardiography images for each group revealed that NOB alleviated the deterioration of cardiac function after MI (*n* = 7, 8, 6, and 8). (d) The cardiac parameters LVEF, LVFS, LVIDs, and LVIDd illustrated the protection of NOB against chronic heart failure after MI (*n* = 7, 8, 6, and 8). (e) The heart weight to body weight ratio decreased after NOB intervention compared with that in the MI group (*n* = 7, 8, 6, and 8). (f) Mice were administrated with NOB or vehicle for 3 days after MI surgery, and Evans blue and TTC staining was performed to identify whether NOB protects against acute heart failure after MI. The results showed no differences between the INF/AAR (infarct area/area at risk) and AAR/LV (area at risk/whole left ventricle area) ratios between the MI+vehicle and MI+NOB groups, indicating that NOB administration could not protect mice after acute MI (the white area indicates the infarct area, the red area shows ischemia, and the blue area indicates normal cardiac tissue) (*n* = 6 and 6). Data are presented as mean ± SD. ^∗∗^*P* < 0.01; ^∗∗∗^*P* < 0.001; ^ns^*P* > 0.05. NOB: nobiletin; MI: myocardial infarction; EF: ejection fraction; FS: fractional shortening; LVIDd: left ventricular internal diameter at end diastole; LVIDs: left ventricular internal diameter at end systole; TTC: triphenyl tetrazolium chloride.

Studies examining the effects of the traditional Chinese medicine qiliqiangxin capsule have revealed cardiac-protective functions mediated via PPAR*γ* regulation [8]. *Citri reticulatae* Pericarpium (CRP), a component of the qiliqiangxin capsule, inhibits pathological cardiac hypertrophy caused by multiple factors [9, 10]. Nobiletin (NOB) is the active monomer isolated from CRP, with the molecular formula C_21_H_22_O_8_ (the chemical structure is shown in Figure 1(a)). NOB has been reported to possess anti-inflammatory [11], antiapoptotic [12], and antineurotoxic [13] functions. In the cardiovascular system, NOB has been reported to attenuate adverse cardiac remodeling in rats following MI by restoring autophagy flux [14] and regulating c-Jun N-terminal kinase (JNK) [15]. However, both the researches mainly focused on rats and the underlying mechanisms through which NOB protects against pathological cardiac remodeling after MI have not been fully explored.

With the use of the HERB database [16] (http://herb.ac.cn/), peroxisome proliferator-activated receptors (PPARs) are potential targets of NOB. PPARs consist of PPAR*α*, PPAR*β*/*δ*, and PPAR*γ*. The PPAR superfamily acts as ligand-inducible transcription factors that regulate various biological processes [17]. Among them, PPAR*γ* binds to PPAR*γ* coactivator 1*α* (PGC1*α*) to form a complex that activates multiple transcription factors and regulates several metabolic processes associated with cardiovascular diseases [18]. Studies have found that ligand-activated PPAR*γ* decreases the inflammatory response in endothelial cells following arterial lesions [19] and attenuates cardiac remodeling in rat hearts following pressure overload [20]. By contrast, the downregulation of PPAR*γ* results in heart failure [21]. Therefore, it is essential to investigate its connection with NOB in the post-MI intervention period, as well as its downstream effectors.

In the present study, we observed that NOB could attenuate pathological cardiac remodeling following MI via the upregulation of PPAR*γ* and PGC1*α*. Abundant *in vivo* and *in vitro* experimental evidence suggests that NOB may have applications for future clinical use.

## 2. Materials and Methods

### 2.1. Animal Preparation

Male C57BL/6 mice (8–10 weeks, 24–26 g) were acquired from Beijing Vital River Laboratory Animal Technology Corporation. All mice were maintained in a temperature- (23 ± 2°C) and humidity-controlled (50% ± 5%) animal center with a 12 h light and 12 h dark cycle under specific pathogen-free (SPF) conditions.

All experiment procedures in this study were approved by the ethical committees of the Nanjing Medical University (IACUC-1803016) and were performed in accordance with the Guide for the Care of Laboratory Animals published by the US National Institutes of Health (NIH publication no. 85-23, revised in 1996).

### 2.2. Establishment of the MI Model

Mice were intraperitoneally anaesthetized with 3% sodium pentobarbital and fixed in a supine position to perform tracheotomy. Body temperatures were maintained within a normal range of 35–37°C, and the mice were artificially ventilated at 120 strokes per minute using a volume-controlled small animal ventilator (Visual Sonic, Canada). A horizontal incision was made along the third to fourth intercostal spaces to expose the heart. In the MI group, the left anterior descending coronary artery (LAD) was ligated 2 mm from the tip of the left auricle using a 7–0 silk suture. The myocardium below the ligation turned pale, and local myocardial movement weakened, indicating the successful establishment of the MI model. For the sham operation group, we crossed the silk under the LAD without performing ligation. After surgery, mice were allocated in an insulation mat for natural awakening.

### 2.3. *In Vivo* Experimental Design

NOB was purchased from Nanjing Ben Cao Co. Ltd. (Nanjing, China), and its purity was >98%, as determined by high-performance liquid chromatography analysis. NOB was dissolved in normal saline containing 0.05% Tween-80 (Sigma, USA). The PPAR*γ* inhibitor (T0070907) was acquired from Selleck Chemicals (St Louis, USA) and dissolved in dimethyl sulfoxide.

To evaluate the anti-CHF protective functions of NOB after MI, 39 experimental mice were randomly assigned into four groups (*n* = 7, 8, 12, and 12, respectively): sham+vehicle, sham+NOB, MI+vehicle, and MI+NOB. Three days after MI surgery, normal saline or NOB (50 mg/kg/d, the dose was as previously described [22]) was administered intragastrically once daily for 3 weeks.

To assess whether NOB could protect cardiac function after acute MI (AMI), 12 mice were randomly divided into two groups (*n* = 6 each): MI+vehicle and MI+NOB. Normal saline or NOB (50 mg/kg/d) was administrated by gavage to mice starting immediately after surgery, once daily for 3 days.

To explore whether NOB exerts cardiac-protective effects following MI via PPAR*γ* upregulation, another *in vivo* experiment was performed, involving 42 experimental mice that were randomly divided into four groups (*n* = 8, 12, 10, and 12, respectively): sham+vehicle, MI+vehicle, MI+NOB, and MI+NOB+PPAR*γ* inhibitor. Vehicle or NOB was administered as described above, and the PPAR*γ* inhibitor was intraperitoneally (1 mg/kg/d) injected into mice once daily for 3 weeks.

Throughout the experimental process, the conditions (survival or death) of the mice were recorded, and a survival curve was generated to further illustrate the survival rate.

### 2.4. Echocardiography

Transthoracic echocardiography was performed to assess the left ventricular function using a Vevo 2100 (Visual Sonics Inc., Toronto, Ontario, Canada) equipped with a 35 MHz transducer before the mice were sacrificed. After the induction of anaesthesia with 1.5%–2% isoflurane, the two-dimensional M-model images were observed from the level of the midpapillary muscle, and cardiac parameters were recorded, including the left ventricular ejection fraction (LVEF), left ventricular fractional shortening (LVFS), left ventricular internal diameter at end diastole (LVIDd), and left ventricular internal diameter at end systole (LVIDs).

### 2.5. Heart Harvesting

After echocardiography, the body weights of mice were measured, and the mice were sacrificed. The hearts were perfused with phosphate-buffered saline (PBS) after thorough thoracic exposure, and the hearts were harvested with the exclusion of the pericardium, thymus, and adjacent organs. The hearts were weighed, and the weights were recorded. The hearts were then fixed with 4% paraformaldehyde solution or frozen in liquid nitrogen.

### 2.6. Haematoxylin and Eosin (HE) Staining and Wheat Germ Agglutinin (WGA) Staining

The harvested hearts were fixed with 4% paraformaldehyde solution. After paraffin embedding, the hearts were sliced into 5 *μ*m thick sections. To evaluate the myocyte sizes, the slides were stained with haematoxylin and eosin (HE) and fluorescein-conjugated wheat germ agglutinin (WGA). The HE and WGA slides were digitally scanned and observed using CaseViewer software. At least ten fields of view were randomly captured for each HE-stained and WGA-stained section, and ImageJ software was used to calculate the myocardial cross-sectional area and evaluate morphological changes and cardiomyocyte sizes.

### 2.7. Masson's Trichrome (Masson) Staining

Masson's trichrome (Masson) staining was performed to measure heart fibrosis. The peri-infarct area of the heart was evaluated with Masson staining. After staining, the slides were scanned and Image-Pro Plus 6.0 (Maryland, USA) software was used to calculate the degree of cardiac fibrosis (the ratio of the fibrotic area to total myocardial area).

### 2.8. Evans Blue and Triphenyl Tetrazolium Chloride (TTC) Staining

To detect the effects of NOB on AMI, mice were sacrificed 3 days after the completion of the NOB intervention, and 1 ml Evans blue (10 mg/ml, dissolved in PBS, Sigma, USA) was slowly injected into the abdominal aorta. After injection, the heart was immediately removed, and infarct size was determined with TTC staining (10 mg/ml, dissolved in PBS, Sigma, USA). The ratio of infarct area (INF) to area at risk (AAR) and the ratio of AAR to left ventricle (LV) area were calculated using ImageJ (NIH) based on five sections throughout the heart.

### 2.9. Neonatal Rat Ventricular Myocyte (NRVM) Isolation

Neonatal Sprague-Dawley rats were obtained from the Experimental Animal Center of Nanjing Medical University with ethical approval. Neonatal rat ventricular monocytes (NRVMs) were isolated from the left ventricles by digestion with trypsin and collagenase type II, followed by Percoll centrifugation as previously described [23]. NRVMs were plated onto gelatine-coated culture dishes in high-glucose Dulbecco's modified Eagle's medium (DMEM, Gibco, Pasadena, CA, USA) with 10% horse serum (HS, Gibco, Carlsbad, CA, USA) and 5% foetal bovine serum (FBS, Gibco, Carlsbad, CA, USA) for 24 h.

### 2.10. *In Vitro* Experiment Design

To investigate the effects of NOB on the alleviation of pathological cardiac hypertrophy, the NRVM medium was replaced with serum-free DMEM to starve the NRVMs for 12 h. NRVMs were subsequently treated with phenylephrine (PE, 100 *μ*M; Sigma, Milwaukee, USA) or NOB (20 *μ*M) for 48 h, followed by harvesting. To further explore whether PPAR*γ* mediates the protective effects of NOB against cardiac hypertrophy induced by PE *in vitro*, NRVMs were separated into the following groups: PE, PE+NOB, PE+NOB+PPAR*γ* inhibitor (T0070907), and PE+NOB+PPAR*γ* agonist (rosiglitazone). The reagent doses were as follows: 100 *μ*M PE, 20 *μ*M NOB, 1 *μ*M T0070907, and 1 *μ*M rosiglitazone.

To examine the effects of NOB on apoptosis, NRVMs were divided into four groups: control, NOB, hypoxia, and hypoxia+NOB. The NRVMs in all groups were cultured in serum-free DMEM for 8 h. The hypoxia and hypoxia+NOB groups were treated with an ischemic buffer, and simulated ischemia was performed in a humidified cell culture incubator (5% O_2_, 95% CO_2_, 37°C) for 8 h. To perform a functional gain and loss experiment, the PPAR*γ* inhibitor and agonist were used, and the groups were divided as follows: hypoxia, hypoxia+NOB, hypoxia+NOB+PPAR*γ* inhibitor, and hypoxia+NOB+PPAR*γ* agonist. NRVMs were harvested at the end of all experiments.

### 2.11. Immunofluorescence Staining

At the end of cultivation, we discarded the medium and washed NRVMs three times with PBS (1×, pH 7.2–7.4), followed by fixation in 4% paraformaldehyde for 20 min. Cells were then permeabilised with 0.2% Triton X-100 in PBS for 30 min and blocked with 10% goat serum in PBS for 1 h. NRVMs were incubated with a mouse *α*-actinin monoclonal antibody (1 : 200, Sigma-Aldrich, St. Louis, MO, USA) at 4°C overnight. After three washes with PBS, NRVMs were incubated with an FITC-labelled secondary IgG antibody (1 : 200, Jackson, USA) at 37°C in the dark for 2 h. Finally, 4′,6-diamidino-2-phenylindole (DAPI, 1 : 100, Sigma-Aldrich, St. Louis, USA) was used to label the nuclei. Cell images were captured using a fluorescence microscope (Carl Zeiss, Oberkochen, Germany). For each group, at least 1,000 cardiomyocytes were counted, and the cell surface area was analysed using ImageJ software.

### 2.12. TUNEL Staining

The terminal deoxynucleotide transferase dUTP nick-end labelling (TUNEL) assay was used to detect the rate of cardiomyocyte apoptosis. NRVMs and peri-infarct cardiac tissue were prepared as described for immunofluorescence staining. After labelling with a secondary antibody, a TUNEL assay kit (Promega, USA) was used to stain apoptotic nuclei, according to the manufacturer's instructions.

### 2.13. Quantitative Reverse Transcription Polymerase Chain Reaction (qRT-PCR)

To detect relative mRNA expression levels in cardiac tissues and NRVMs, the TRIzol reagent was used to extract total RNA, according to the manufacturer's protocol (Takara, Japan). Reverse transcription was performed using 500 ng total RNA to synthesise the complementary DNA (cDNA) using the iScript™ cDNA Synthesis Kit (Bio-Rad, Hercules, CA, USA). Using the appropriate proportions of the SYBR Green qPCR Master Mix kit (Bio-Rad, Hercules, CA, USA), cDNA, and deionised water, the ABI-7900 Real-Time PCR Detection System (7900HT, Applied Biosystems, CA, USA) was used to quantify mRNA expression, which was normalised against the housekeeping gene glyceraldehyde 3-phosphate dehydrogenase (GAPDH) using the comparative quantification method (2^−ΔΔCT^). All primers utilised for amplification are listed in Supplementary Table 1.

### 2.14. Western Blot (WB)

Total protein from the left ventricular tissues or cultured NRVMs was collected using radioimmunoprecipitation assay (RIPA) lysis buffer (P0013C, Beyotime, Shanghai, China) supplemented with 1 mM phenylmethylsulfonyl fluoride (PMSF, ST505, Beyotime, Shanghai, China). Equivalent protein quantities were separated by 10% sodium dodecyl sulfate-polyacrylamide gel electrophoresis (SDS-PAGE) and transferred onto polyvinylidene difluoride (PVDF) membranes (Millipore, Billerica, MA, USA). After blocking nonspecific binding sites with 5% nonfat dried milk powder for 2 h at room temperature, the membrane was incubated with primary antibodies overnight at 4°C. After incubation with secondary antibodies (room temperature for 2 h), the intensity of the protein bands was visualised by using Labworks software (Bio-Rad, USA) and analysed by using ImageJ software. The primary antibodies used in this study were listed as follows: proliferator-activated receptor gamma (PPAR*γ*, 1 : 1,000, Proteintech Group, Wuhan, China), PPAR*γ* coactivator 1-alpha (PGC1*α*, 1 : 1,000, Novus Biologicals, Littleton, Colorado, USA), B-cell lymphoma 2 (Bcl-2, 1 : 1,000, Cell Signaling Technology, Boston, Massachusetts, USA), Bcl-2-associated X protein (Bax, 1 : 1000, Cell Signaling Technology, Boston, Massachusetts, USA), collagen type 1 (collagen 1, 1 : 1000, Proteintech Group, Wuhan, China), *α*-smooth muscle actin (*α*-SMA, 1 : 1000, Proteintech Group, Wuhan, China), cleaved caspase-3 (CC3, 1 : 1000, Cell Signaling Technology, Boston, Massachusetts, USA), nuclear factor erythroid 2-related factor 2 (Nrf-2, 1 : 1000, Abcam, Cambridge, UK), heme oxygenase 1 (HO-1, 1 : 1000, Abcam, Cambridge, UK), *β*-tubulin (1 : 1000, Bioworld Technology, Minnesota, USA), and glyceraldehyde 3-phosphate dehydrogenase antibody (GAPDH, 1 : 1000, Kangchen, Shanghai, China).

### 2.15. Statistical Analysis

All data are expressed as the mean ± standard deviation (SD). All statistical analyses were performed using GraphPad Prism 6.0 Software. Statistical comparisons among multiple groups were examined using one-way analysis of variance (ANOVA) followed by Bonferroni's post hoc test, and differences between two groups were analyzed using an independent sample *t*-test. A difference of *P* < 0.05 was considered significant.

## 3. Results

### 3.1. NOB Protects against CHF after MI

To identify the protective effects of NOB against CHF after MI, we established an MI mouse model. NOB or vehicle was intragastrically administered to mice for 3 weeks, starting 3 days after surgery. The numbers of mice for the sham+vehicle, sham+NOB, MI+vehicle, and MI+NOB groups were 7, 8, 12, and 12, respectively, at the start of the experiment, and after 3 weeks, the numbers were 7, 8, 6, and 8, respectively. Six mice were sacrificed in the MI+vehicle group, and four were sacrificed in the MI+NOB group. The survival rates for each group were 100%, 100%, 50%, and 66.7%, showing that NOB increased the survival rate among CHF mice after MI (Figure 1(b)). As shown by echocardiography, compared with the sham group, the LVEF and LVFS were significantly decreased in the MI group, and the LVIDd and LVIDs were enlarged. Following NOB intervention, cardiac function and cardiac parameters improved remarkably (Figures 1(c) and 1(d)). Moreover, the heart weight/body weight ratio increased in the MI group compared with that in the NOB intervention group, confirming that decompensated hypertrophy was alleviated after NOB intervention (Figure 1(e)).

After establishing the MI mouse model, NOB or vehicle was immediately administrated and lasted for 3 days. Evans blue and TTC staining was performed to evaluate the infarcted and ischemic area. The results illustrated that the INF/AAR and AAR/LV ratios were not significantly different between the MI+vehicle and MI+NOB group (*P* > 0.05), indicating that NOB intervention failed to protect against acute heart failure after AMI (Figure 1(f)).

### 3.2. NOB Mitigates Pathological Cardiac Remodeling after MI

**Figure 2:**
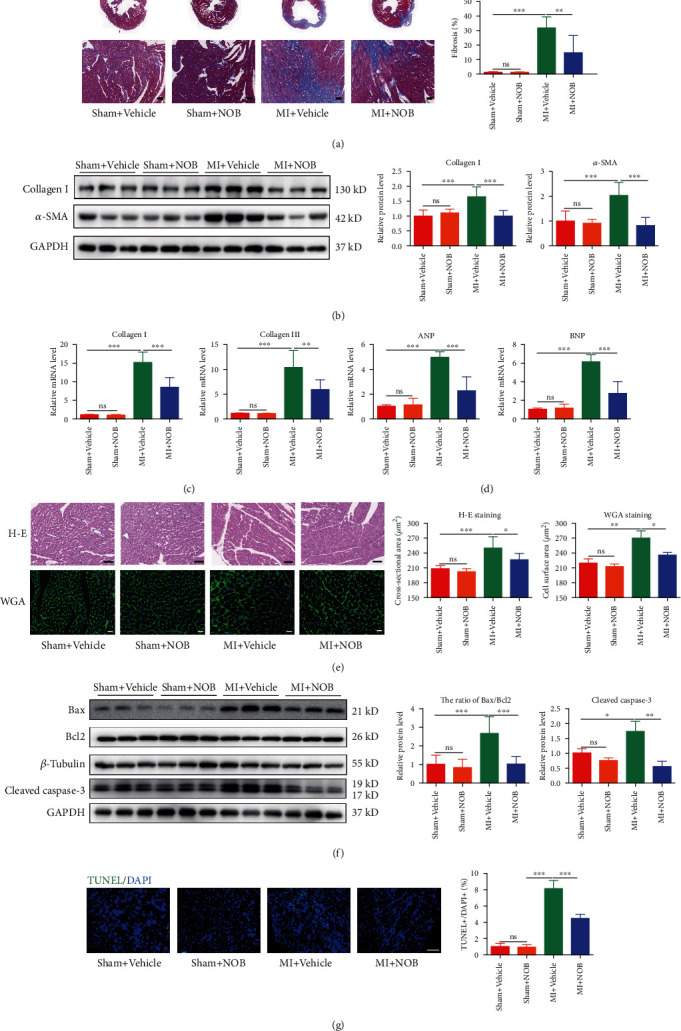
NOB mitigates pathological cardiac remodeling after MI. (a) Masson's trichrome staining indicated that the fibrosis rate of the peri-infarct area descended significantly after NOB intervention (blue, fibrosis-positive area; red, fibrosis-negative area; scale bars: 50 *μ*m) (*n* = 7, 8, 6, and 8). (b) Western blot analysis revealed that collagen I and *α*-SMA were dramatically decreased in NOB intervention mice after MI (*n* = 6 per group). (c) qRT-PCR analysis of collagen I and collagen III showed that NOB alleviated cardiac fibrosis after MI (*n* = 6 per group). (d) qRT-PCR analysis showed that NOB alleviated the upregulation of ANP and BNP induced by MI (*n* = 6 per group). (e) HE staining revealed that the cross-sectional areas of cardiomyocytes were smaller in the intervention group than in the MI group (scale bars: 50 *μ*m, *n* = 7, 8, 6, and 8). WGA staining illuminated the same trends as HE staining: the increased cross-sectional areas of cardiomyocytes were smaller after NOB intervention (scale bars: 20 *μ*m, *n* = 3 per group). (f) Western blot showed that the ratio of Bax/Bcl2 and the expression of cleaved caspase-3 were both lower in the NOB intervention group than in the MI group (*n* = 6 per group for Bax/Bcl2 and *n* = 3 per group for cleaved caspase-3). (g) TUNEL staining displayed that the upregulated apoptotic-positive cardiomyocyte rate was decreased after NOB intervention in cardiac tissue (blue: nuclei; green: apoptotic-positive nuclei; scale bars: 100 *μ*m, *n* = 3 per group). Data are presented as mean ± SD. ^∗^*P* < 0.05; ^∗∗^*P* < 0.01; ^∗∗∗^*P* < 0.001; ^ns^*P* > 0.05. NOB: nobiletin; MI: myocardial infarction; ANP: natriuretic peptide type A; BNP: natriuretic peptide B; HE staining: haematoxylin and eosin staining; WGA staining: wheat germ agglutinin; TUNEL: terminal deoxynucleotidyl transferase-mediated dUTP-biotin nick end labeling.

Masson staining of the peri-infarct area of the heart revealed a significantly increased fibrosis rate in MI mice, which was decreased by NOB administration (Figure 2(a)). Western blot (WB) was used to evaluate the protein levels of classical fibrosis markers, which showed that collagen I and *α*-smooth muscle actin (*α*-SMA) increased prominently in cardiac tissue after MI, which decreased after NOB intervention (Figure 2(b)). Similarly, the expression of collagen I and collagen III in mRNA level showed the same trends as the protein (Figure 2(c)). These results suggested that NOB could alleviate myocardial fibrosis after MI.

Classical pathological cardiac hypertrophy biomarkers, including atrial natriuretic peptide (ANP) and brain natriuretic peptide (BNP), were upregulated in the MI group and reduced in the NOB intervention group (Figure 2(d)). The HE and WGA staining results showed that the cross-sectional area of cardiomyocytes was larger in the MI group, and these reduced in size following NOB administration (Figure 2(e)). These results further suggested that NOB might alleviate decompensated cardiac hypertrophy after MI.

WB was used to detect changes in apoptotic protein expression. The Bax/Bcl2 ratio and the expression of cleaved caspase-3 were prominently increased in MI cardiac tissue and could be reduced by NOB intervention (Figure 2(f)). Moreover, the apoptosis-positive cardiomyocyte rate was elevated in the MI group compared with the sham group but decreased after NOB intervention for 3 weeks, as revealed from TUNEL staining (Figure 2(g)). Collectively, these results indicated that NOB functions as a negative regulator of cardiac apoptosis.

### 3.3. NOB Attenuates Hypoxia-Induced Apoptosis and PE-Induced Cardiac Hypertrophy in NRVMs

**Figure 3:**
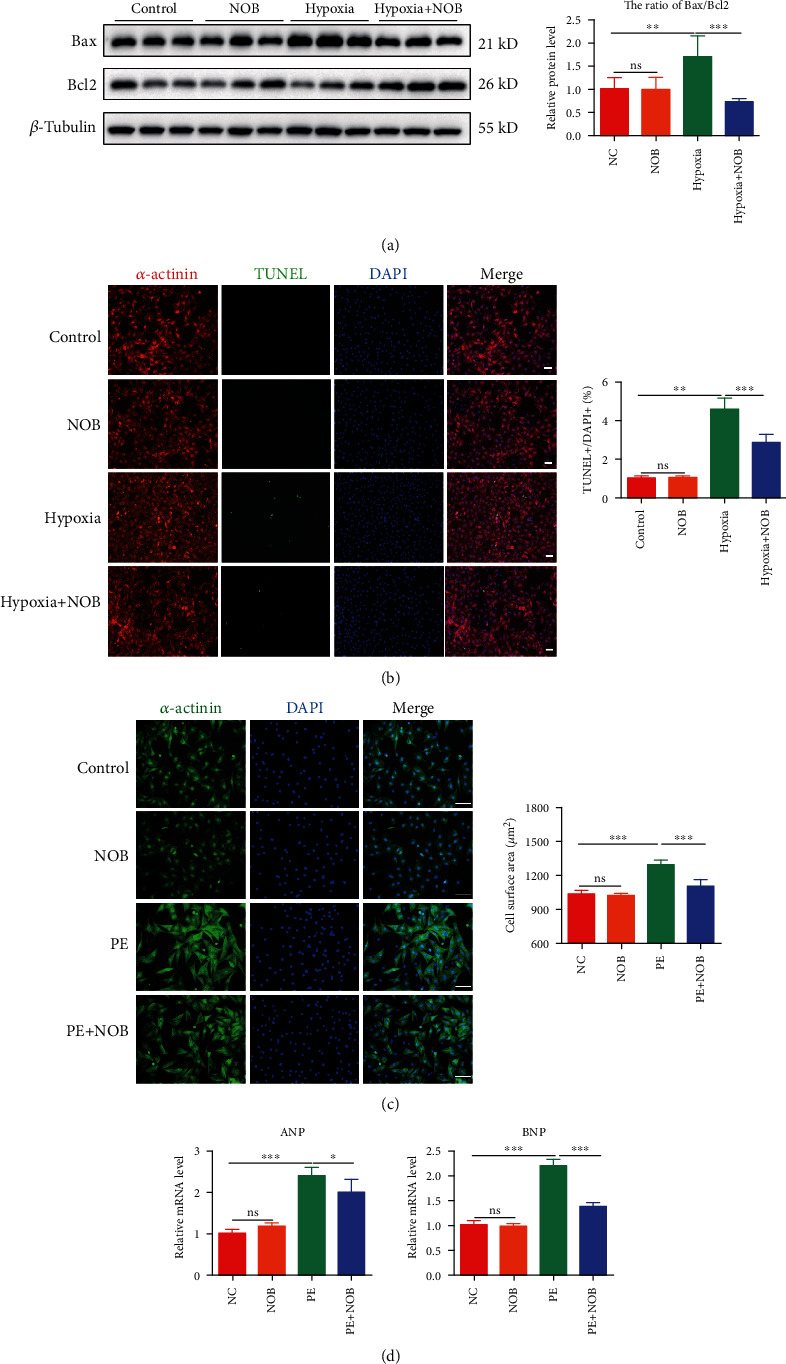
NOB attenuates hypoxia-induced apoptosis and PE-induced cardiac hypertrophy in NRVMs. (a) Western blot analysis of Bax and Bcl2 indicated that the Bax/Bcl2 ratio increased after hypoxia and decreased with NOB administration (*n* = 6 per group). (b) TUNEL staining indicated that the rate of apoptosis-positive NRVMs was reduced after NOB interference compared with that in the hypoxia group (blue: nuclei; red: *α*-actinin; and green: apoptotic-positive nuclei; scale bars: 100 *μ*m, *n* = 6 per group). (c) Immunofluorescence staining in NRVMs (blue: nuclei; green: *α*-actinin; scale bars: 100 *μ*m) showed that the NRVM cell size increased in the PE group compared with the control group, which could be reversed by NOB intervention (*n* = 6 per group). (d) qRT-PCR showed that the expression levels of ANP and BNP were lower in the NOB intervention group than in the PE-induced group (*n* = 6 per group). Data are presented as mean ± SD. ^∗^*P* < 0.05; ^∗∗^*P* < 0.01; ^∗∗∗^*P* < 0.001; ^ns^*P* > 0.05. NOB: nobiletin; NRVMs: neonatal rat ventricular cardiomyocytes; PE: phenylephrine; TUNEL: terminal deoxynucleotidyl transferase-mediated dUTP-biotin nick end labeling; ANP: natriuretic peptide type A; BNP: natriuretic peptide B.

To evaluate the function of NOB in NRVMs, we first constructed a hypoxia-induced apoptosis model. The ratio of the apoptotic biomarkers Bax/Bcl2 was significantly elevated in the hypoxia group, whereas NOB intervention reduced the ratio (Figure 3(a)). TUNEL staining was used to specifically evaluate apoptotic NRVMs. After hypoxia, the TUNEL-positive rate increased by 5%, which could be halved by NOB intervention (Figure 3(b)). These results further indicated that NOB mitigates hypoxia-induced apoptosis.

To appraise the function of NOB in pathological cardiac hypertrophy, we used PE to construct an NRVM hypertrophy model. After calculating cell sizes using immunofluorescence staining, the enlarged cell sizes in the PE group were conspicuously decreased after NOB administration (Figure 3(c)). The MI-induced upregulation of ANP and BNP, as determined by qRT-PCR, decreased with NOB intervention (Figure 3(d)). These results indicated that NOB ameliorated PE-induced pathological cardiac hypertrophy.

### 3.4. NOB Activates PPAR*γ* and PGC1*α* In Vivo and In Vitro

**Figure 4:**
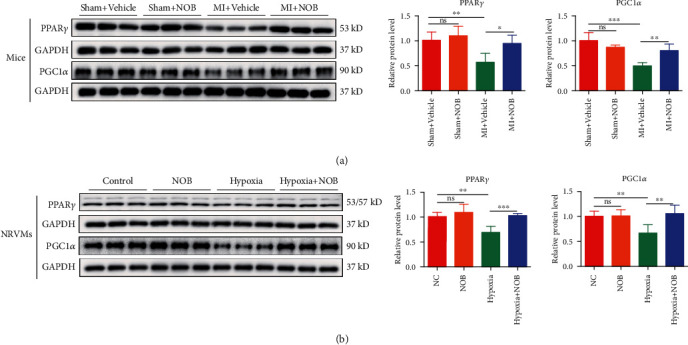
NOB activates PPAR*γ* and PGC1*α in vivo* and *in vitro*. (a) In mouse hearts, PPAR*γ* and PGC1*α* expression significantly decreased in the MI group, which was reversed by NOB intervention (*n* = 6 per group). (b) The expression levels of PPAR*γ* and PGC1*α* were higher in the NOB intervention group than in the hypoxia group (*n* = 6 per group). Data are presented as mean ± SD. ^∗^*P* < 0.05; ^∗∗^*P* < 0.01; ^∗∗∗^*P* < 0.001; ^ns^*P* > 0.05. NOB: nobiletin; PPAR*γ*: peroxisome proliferator-activated receptor gamma; PGC1*α*: PPAR*γ* coactivator 1*α*; MI: myocardial infarction; NRVMs: neonatal rat ventricular cardiomyocytes.

Alterations in PPAR*γ* and PGC1*α* expression have been found to play vital roles in the regulation of energy metabolism. The expression levels of PPAR*γ* and PGC1*α* were examined by WB, which showed that the expression levels in cardiac tissue (Figure 4(a)) and NRVMs (Figure 4(b)) were consistent. Damage to cardiac tissue or NRVMs resulted in PPAR*γ* and PGC1*α* downregulation, whereas NOB intervention could reverse these trends, confirming that PPAR*γ* and PGC1*α* might be involved in the specific mechanism through which NOB protects against pathological cardiac remodeling after MI.

### 3.5. PPAR*γ* Inhibitor (T0070907) Weakened the Protective Effects of NOB against Hypoxia-Induced Apoptosis and PE-Induced Pathological Cardiac Hypertrophy in NRVMs

**Figure 5:**
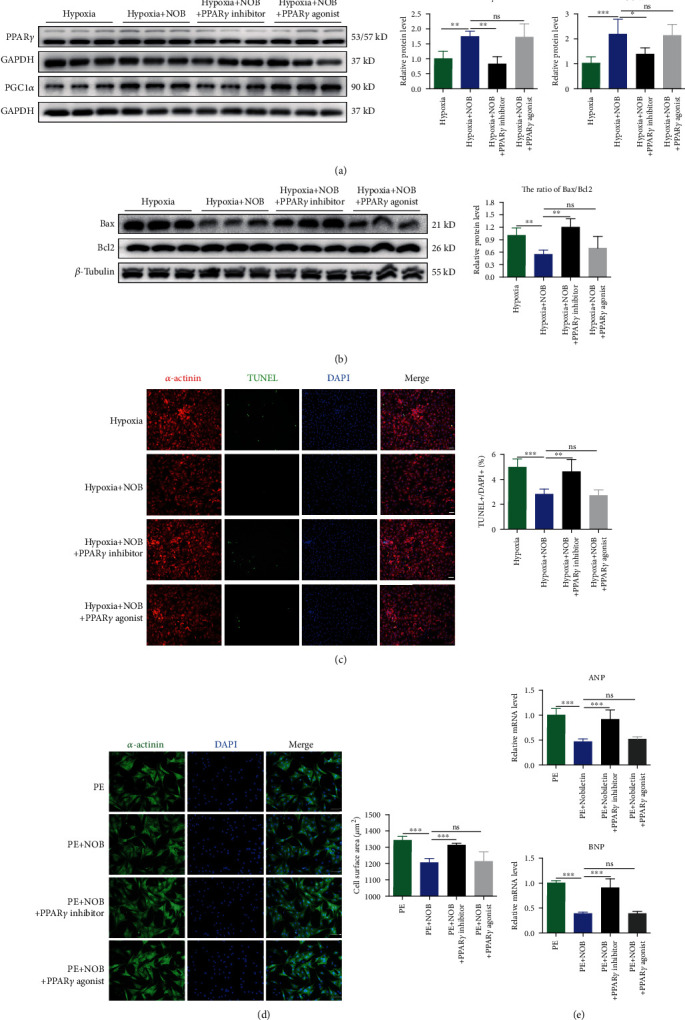
PPAR*γ* inhibitor (T0070907) weakened the protective effects of NOB against hypoxia-induced apoptosis and PE-induced pathological cardiac hypertrophy in NRVMs. (a) In the hypoxia-induced apoptotic model, the expression levels of PPAR*γ* and PGC1*α* were downregulated in the PPAR*γ* inhibitor group compared with the NOB intervention group, confirming the inhibitor function (*n* = 6 per group). (b) In the hypoxia model, the combined intervention of NOB and PPAR*γ* inhibitor (T0070907) increased the Bax/Bcl2 ratio compared with NOB intervention alone, whereas cointervention with both the NOB and PPAR*γ* agonist (rosiglitazone) resulted in a Bax/Bcl2 ratio similar to that of the NOB group (*n* = 6 per group). (c) TUNEL staining showed reduced apoptosis in NRVMs in the NOB intervention group, but this effect was eliminated by the PPAR*γ* inhibitor (blue: nuclear; red: *α*-actinin; and green: apoptotic-positive nuclear; scale bars: 100 *μ*m, *n* = 6 per group). (d) The results of immunofluorescence staining showed that the cell sizes of NRVMs were larger in the PPAR*γ* inhibitor group, whereas PPAR*γ* agonists failed to further shrink the cell size (blue: nuclear; green: *α*-actinin; scale bars: 100 *μ*m, *n* = 6 per group). (e) The expression of ANP and BNP increased in the PPAR*γ* inhibitor group (*n* = 6 per group). Data are presented as mean ± SD. ^∗^*P* < 0.05; ^∗∗^*P* < 0.01; ^∗∗∗^*P* < 0.001; ^ns^*P* > 0.05. PPAR*γ*: peroxisome proliferator-activated receptor gamma; NOB: nobiletin; NRVMs: neonatal rat ventricular cardiomyocytes; TUNEL: terminal deoxynucleotidyl transferase-mediated dUTP-biotin nick end labeling; PGC1*α*: PPAR*γ* coactivator 1*α*; ANP: natriuretic peptide type A; BNP: natriuretic peptide B.

The downregulation of PPAR*γ* and PGC1*α* after MI could be reversed by NOB administration. Therefore, we examined whether PPAR*γ* regulation is essential for the protective effects of NOB against pathological cardiac remodeling after MI by performing functional gain and loss experiments in NRVMs using both a PPAR*γ* inhibitor and an agonist. The results indicated that the upregulated expression of PPAR*γ* and PGC1*α* in the intervention group were downregulated after the administration of the PPAR*γ* inhibitor detected by WB (Figure 5(a)). The NOB-induced reduction in the ratio between the apoptotic-related proteins, Bax/Bcl2, was reversed in the presence of the PPAR*γ* inhibitor; however, the agonist failed to enhance the reduction of apoptosis, and the Bax/Bcl2 ratios were similar between the NOB intervention and agonist groups (Figure 5(b)). TUNEL staining was used to calculate the NRVM apoptosis rate, which showed that the PPAR*γ* inhibitor upregulated the TUNEL-positive rate compared with the intervention group. By contrast, no difference was observed between the intervention and agonist groups (Figure 5(c)). When we repeated the pathological hypertrophy experiment, the decreased cell size (Figure 5(d)) and the downregulated expression levels of ANP and BNP (Figure 5(e)) observed following NOB intervention were reversed by the presence of the PPAR*γ* inhibitor. These results indicated that the PPAR*γ* inhibitor eliminated the protective effects of NOB against apoptosis and cardiac hypertrophy *in vitro*.

### 3.6. PPAR*γ* Inhibitor (T0070907) Reverses the Protective Effects of NOB on CHF after MI

**Figure 6:**
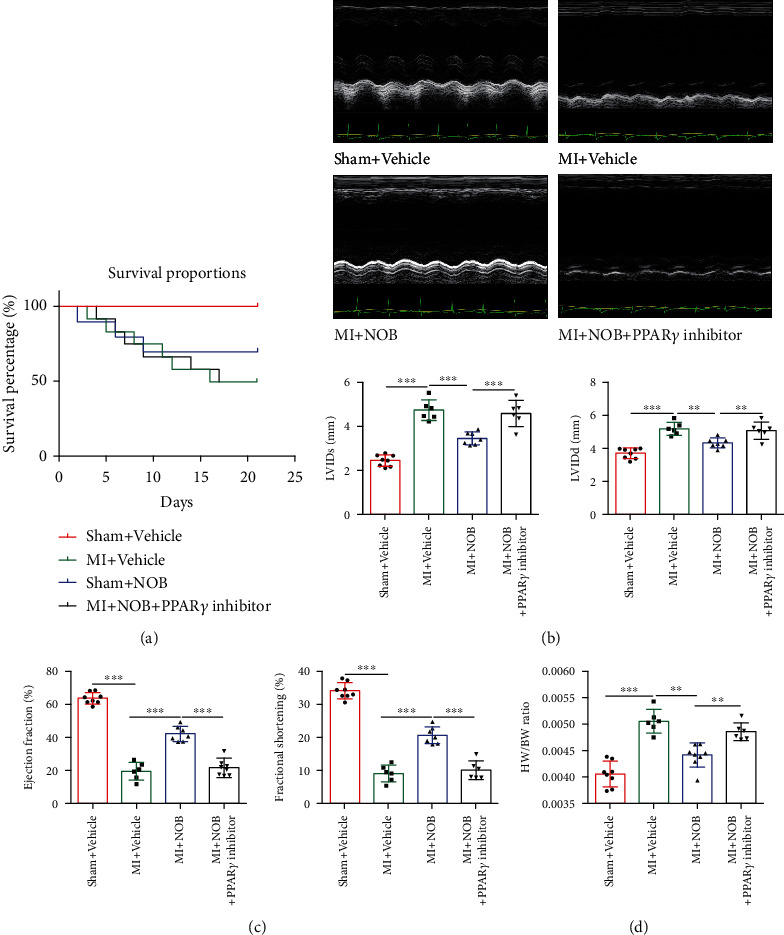
PPAR*γ* inhibitor (T0070907) reversed the protective effects of NOB against chronic heart failure after MI. (a) The survival curve showed that the mortality rate of the PPAR*γ* inhibitor group was significantly higher than that of the NOB intervention group. The numbers of mice for the sham+vehicle, MI+vehicle, MI+NOB, and MI+NOB+PPAR*γ* inhibitor were 8, 12, 10, and 12 at the beginning of the experiment, whereas after 3 weeks, the numbers were 8, 6, 7, and 6, respectively. Six mice were sacrificed in the MI+vehicle and MI+NOB+PPAR*γ* inhibitor group, and four were sacrificed in the MI+NOB group. The survival rates for each group were 100%, 50%, 70%, and 50%. (b) Representative echocardiography images for all four groups (*n* = 8, 6, 7, and 6). (c) Parameters reflecting cardiac function showed reduced LVEF and LVFS and dilated LVIDd and LVIDs following PPAR*γ* inhibitor administration, revealing worsened cardiac function after PPAR*γ* inhibitor injection (*n* = 8, 6, 7, and 6). (d) The heart weight/body weight ratio increased in the inhibitor group (*n* = 8, 6, 7, and 6). Data are presented as mean ± SD. ^∗∗^*P* < 0.01; ^∗∗∗^*P* < 0.001. PPAR*γ*: peroxisome proliferator-activated receptor gamma; NOB: nobiletin; MI: myocardial infarction; LVEF: left ventricular ejection fraction; LVFS: left ventricular fractional shortening; LVIDd: left ventricular internal diameter at end diastole; LVIDs: left ventricular internal diameter at end systole.

Based on the results of the *in vitro* experiment, we further investigated the function of the PPAR*γ* inhibitor in mice. Normal saline or NOB was administrated by gavage starting 3 days after the MI induction surgery and lasting for 3 weeks. The PPAR*γ* inhibitor was intraperitoneally injected once daily and lasted for 3 weeks. Mice in the inhibitor group experienced higher mortality compared with the NOB intervention group based on the survival curve (Figure 6(a)). The echocardiography showed worsened cardiac function in the inhibitor group, including worsened LVEF and LVFS and a dilated left ventricular diameter (Figures 6(b) and 6(c)). The amelioration of decompensated myocardial hypertrophy induced by NOB was eliminated by PPAR*γ* inhibitor administration, resulting in an enhanced HW/BW ratio (Figure 6(d)).

### 3.7. PPAR*γ* Is Essential for the Protective Effects of NOB against Cardiac Remodeling after MI

**Figure 7:**
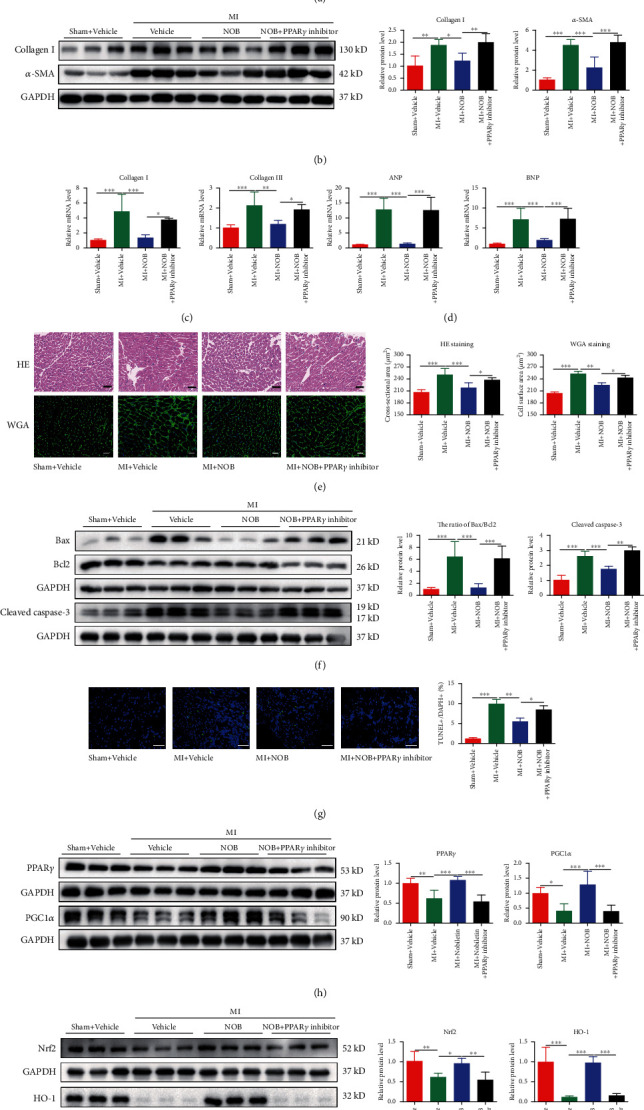
PPAR*γ* is essential for the protective effects of NOB against cardiac remodeling after MI. (a) Masson's trichrome staining of the peri-infarct area indicated increased heart fibrosis following PPAR*γ* inhibitor injection compared with the NOB intervention group (*n* = 8, 6, 7, and 6). (b) The protein expression of collagen I and *α*-SMA was significantly increased after PPAR*γ* inhibitor injection (*n* = 6 per group). (c) Increased mRNA expression levels of collagen I and collagen III also indicated fibrosis (*n* = 6 per group). (d) The expression levels of ANP and BNP were significantly elevated in the inhibitor group (*n* = 6 per group). (e) HE and WGA staining showed the enlargement of the cross-sectional area in the inhibitor group (scale bars: 50 *μ*m and *n* = 8, 6, 7, and 6 for HE staining; scale bars: 20 *μ*m and *n* = 3 per group for WGA staining). (f) The ratio of Bax/Bcl2 and the expression of cleaved caspase-3 were both higher in the inhibitor group than in the NOB intervention group (*n* = 6 per group for Bax/Bcl2 and *n* = 3 per group for cleaved caspase-3). (g) TUNEL staining showed that the apoptosis-positive cardiomyocyte rate was upregulated in the PPAR*γ* inhibitor group compared with the NOB intervention group (scale bars: 100 *μ*m, *n* = 3 per group). (h) The protein expression level of PPAR*γ* and PGC1*α* decreased after PPAR*γ* inhibitor injection (*n* = 6 per group). (i) WB analysis showed that the expression of Nrf-2 and HO-1 was downregulated in the MI group and upregulated following NOB intervention; the PPAR*γ* inhibitor injection abolished the upregulated expression of Nrf-2 and HO-1 (*n* = 6 per group). Data are presented as mean ± SD. ^∗^*P* < 0.05; ^∗∗^*P* < 0.01; ^∗∗∗^*P* < 0.001. PPAR*γ*: peroxisome proliferator-activated receptor gamma; NOB: nobiletin; ANP: natriuretic peptide type A; BNP: natriuretic peptide B; HE staining: haematoxylin and eosin staining; WGA staining: wheat germ agglutinin staining; TUNEL: terminal deoxynucleotidyl transferase-mediated dUTP-biotin nick end labeling; PGC1*α*: PPAR*γ* coactivator 1*α*; Nrf2: nuclear factor erythroid 2-related factor 2; HO-1: heme oxygenase 1.

Masson staining of the peri-infarct area of the heart showed an increased fibrosis rate in the inhibitor group compared with the intervention group (Figure 7(a)) and increased biomarker expression, including collagen I and *α*-SMA (Figure 7(b)). The mRNA levels of collagen I and collagen III (Figure 7(c)) remained consistent, indicating that the PPAR*γ* inhibitor could impair the protective effects of NOB against fibrosis. The beneficial effects of NOB against decompensated cardiac hypertrophy were also reversed by the PPAR*γ* inhibitor, as indicated by the enhanced ANP and BNP expression assessed by qRT-PCR (Figure 7(d)) and the enlarged cross-sectional area of cardiomyocytes revealed by HE and WGA staining (Figure 7(e)). The Bax/Bcl2 ratio and cleaved caspase-3 expression level were both upregulated in the PPAR*γ* inhibitor group (Figure 7(f)), and the reduction in apoptotic-positive cardiomyocytes observed in the NOB intervention group was upregulated by PPAR*γ* inhibitor injection (Figure 7(g)), confirming that the PPAR*γ* inhibitor reversed the protective effects of NOB against apoptosis. The WB analysis revealed that the expression of PPAR*γ* and PGC1*α* was downregulated in the PPAR*γ* inhibitor group compared with the NOB intervention group (Figure 7(h)). The elimination of the NOB-mediated protective effect against pathological cardiac remodeling after MI by the PPAR*γ* inhibitor suggested that PPAR*γ* and PGC1*α* are indispensable for the protective effects of NOB against pathological cardiac remodeling after MI. Furthermore, WB analysis revealed that the expression of Nrf-2 and HO-1 was decreased following MI and increased after NOB intervention, whereas PPAR*γ* inhibitor injection reversed this upregulation (Figure 7(i)). Therefore, we speculated that Nrf-2/HO-1 could be the potential downstream effectors of PPAR*γ* in the protective mechanism through which NOB alleviates pathological cardiac remodeling after MI.

## 4. Discussion

Recent strategies for alleviating pathological cardiac remodeling have primarily focused on inhibitors of the angiotensin system, the sympathetic nervous system, and neprilysin [24]. However, the morbidity of pathological cardiac remodeling after MI remains high; thus, the identification of potential therapeutic targets is vital [25].

Nobiletin (NOB) is thought to be the primary active ingredient extracted from CRP. Recent studies have elucidated the protective effects of NOB on the cardiovascular system. NOB attenuates cardiac dysfunction, oxidative stress, and inflammation in a model of diabetic cardiomyopathy induced by streptozotocin [22, 26]. In this study, we found that NOB protects against CHF and pathological cardiac remodeling in mice after MI. NOB also ameliorated apoptosis and hypertrophy in NRVMs induced by hypoxia and PE, respectively.

The mechanisms underlying pathological cardiac remodeling are complicated, and myocardial fibrosis, apoptosis, and decompensated hypertrophy have important effects [27]. Stimulation with neuroendocrine factors or growth factors increases cardiomyocyte protein synthesis, causing myocardial hypertrophy [28, 29]. Simultaneously, increased protein expression in cardiomyocytes causes ER stress, which represents a key regulator of cellular apoptosis [30]. In addition, myocardial fibroblast proliferation is induced through the transforming growth factor *β* (TGF-*β*) pathway, renin-angiotensin-aldosterone system (RAAS) overactivation, and other pathways, triggering the continued release of profibrotic factor [31, 32].

During the early stages of ischemic cardiomyopathy, myocardial energy metabolism is insufficient. PPAR*γ*, together with its coactivator PGC1*α*, is a well-known regulator of cardiac energy homeostasis [18]. Studies have shown that PGC1*α* expression levels are associated with obesity, diabetes, lipid metabolism disorders, and cardiovascular diseases [33]. PPAR*γ* is a ligand-activated transcription factor belonging to the PPAR nuclear receptor family that regulates lipid and glucose metabolism, immune inflammation, cell proliferation, and differentiation [34, 35]. During cardiovascular diseases, the activation of PPAR*γ* reduces myocardial fibrosis [36] and apoptosis [37], improves myocardial ischemia-reperfusion injury [38], and inhibits myocardial hypertrophy [39]. In addition, PPAR*γ* activity regulation has significant clinical applications, as PPAR*γ* agonists have been used as hypoglycaemic drugs [40, 41]. However, whether PPAR*γ* and PGC1*α* mediate the protective effects of NOB against cardiac remodeling after MI remains unclear. In our study, we demonstrated that PPAR*γ* and PGC1*α* were downregulated after MI and upregulated following NOB intervention. Functional gain and loss experiments further demonstrated that PPAR*γ* inhibition could block the protective effects of NOB against pathological cardiac remodeling.

Furthermore, with the validation of the expression of Nrf-2 and HO-1 in cardiac tissue of post-MI mice by WB, we observed that both the expression of Nrf-2 and HO-1 showed the same trends as PPAR*γ* and PGC1*α* in the NOB protective process. Nrf-2 is a pivotal transcriptional factor that regulates the cardiac homeostasis via suppressing oxidative stress. Emerging evidence has confirmed its important function in regulating ischemic heart disease, heart failure, myocardial infarction, atrial fibrillation, and myocarditis [42]. HO-1 has been extensively recognized as the downstream of Nrf-2 and plays a key role in cell adaptation to stressors through the antioxidant, antiapoptotic, and anti-inflammatory properties of its metabolic products [43, 44]. Chen et al. [45] found that polydatin protects against acute myocardial infarction-induced cardiac damage by the activation of Nrf-2/HO-1 signaling. Ulteriorly, Polvani et al. [46] summarized that PPAR*γ* can activate Nrf-2 directly or indirectly via an upstream route. Based on the theory, Wu et al. [47] discovered that overexpression of high mobility group protein AT-hook 2 (HMGA2) ameliorates cardiac remodeling in response to pressure overload via the activation of the PPAR*γ*/Nrf2 signaling pathway. Therefore, we speculated that the antipathological cardiac remodeling effect of NOB after MI was through activating PPAR*γ* and PGC1*α*; the potential downstream effectors of PPAR*γ* in the protective process could be Nrf-2/HO-1.

However, this study has some limitations. First, the use of PPAR*γ*-knockout mice remains necessary to further verify our findings. Second, although PPAR*γ* and PGC1*α* have been identified as potential targets for the protective effects of NOB against pathological cardiac remodeling after MI and we have initially verified that Nrf2/HO-1 could be the potential downstream effectors of PPAR*γ* in the protective process, the interaction between them and whether other downstream effectors exist are worth further investigation.

## 5. Conclusions

In conclusion, our study demonstrated the protective effects of NOB for the alleviation of pathological cardiac remodeling across three dimensions (cardiac fibrosis, apoptosis, and decompensated hypertrophy) following MI in mice. In addition, NOB intervention reduced apoptosis and hypertrophy in NRVMs. This protective effect is mediated by the upregulation of PPAR*γ* and PGC1*α*. Functional gain and loss experiments revealed that the PPAR*γ* inhibitor abolished the protective events of NOB *in vivo* and *in vitro*. Moreover, Nrf-2/HO-1 might serve as the potential downstream effectors of PPAR*γ* in the protective process. Our research suggested that NOB could represent a potential clinical therapeutic treatment for the alleviation of pathological cardiac remodeling after MI.

## Data Availability

The data used to support the findings of our study are available from the co-first authors Yufei Zhou and Ting Yin and the corresponding author Xinli Li upon reasonable request.

## Abbreviations

NOB: Nobiletin
MI: Myocardial infarction
CHF: Chronic heart failure
EF: Ejection fraction
FS: Fractional shortening
LVIDd: Left ventricular internal diameter at end diastole
LVIDs: Left ventricular internal diameter at end systole
TTC: Triphenyl tetrazolium chloride
ANP: Natriuretic peptide type A
BNP: Natriuretic peptide B
NRVMs: Neonatal rat ventricular cardiomyocytes
PE: Phenylephrine
TUNEL: Terminal deoxynucleotidyl transferase-mediated dUTP-biotin nick end labeling
PPAR*γ*: Peroxisome proliferator-activated receptor gamma
PGC1*α*: PPAR*γ* coactivator 1*α*.

## Supplementary Materials

Supplementary Materials Supplementary Table 1: list of primers used in our study. Graphic abstract: nobiletin attenuates pathological cardiac remodeling after myocardial infarction via activating PPAR*γ* and PGC1*α*.
