# Supplementary material for: Nobiletin Attenuates Pathological Cardiac Remodeling after Myocardial Infarction via Activating PPARγ and PGC1α
Source: PPAR Res. 2021 Aug 6;2021:9947656. doi: 10.1155/2021/9947656 (PMC8373512; doi:10.1155/2021/9947656)
Supplement: Supplementary Materials — Supplementary Table 1: list of primers used in our study. Graphic abstract: nobiletin attenuates pathological cardiac remodeling after myocardial infarction via activating PPARγ and PGC1α. [file 9947656.f1.docx]

**Supplementary Table 1.** List of primers used in our study

| Gene | Forward primer | Reverse primer |
| --- | --- | --- |
| Rat-ANP | GAGCAAATCCCGTATACAGTGC | ATCTTCTACCGGCATCTTCTCC |
| Rat-BNP | GCTGCTGGAGCTGATAAGAGAA | GTTCTTTTGTAGGGCCTTGGTC |
| Rat-GAPDH | AAGCTCACTGGCATGGCCTT | CGGCATGTCAGATCCACAAC |
| Mouse-ANP | AGGCAGTCGATTCTGCTT | CGTGATAGATGAAGGCAGGAAG |
| Mouse-BNP | TAGCCAGTCTCCAGAGCAATTC | TTGGTCCTTCAAGAGCTGTCTC |
| Mouse-GAPDH | CCTTCCGTGTTCCTACCCC | GCCCAAGATGCCCTTCAGT |
| Mouse-Collagen I | TCTAGACATGTTCAGCTTTGTGGAC | TCTGTACGCAGGTGATTGGTG |
| Mouse-Collagen III | CTGTAACATGGAAACTGGGGAAA | CCATGACTGAACTGAAAACCACC |

Abbreviations: ANP, atrial natriuretic polypeptide; BNP, brain natriuretic polypeptide; GAPDH, glyceraldehyde 3-phosphate dehydrogenase antibody.


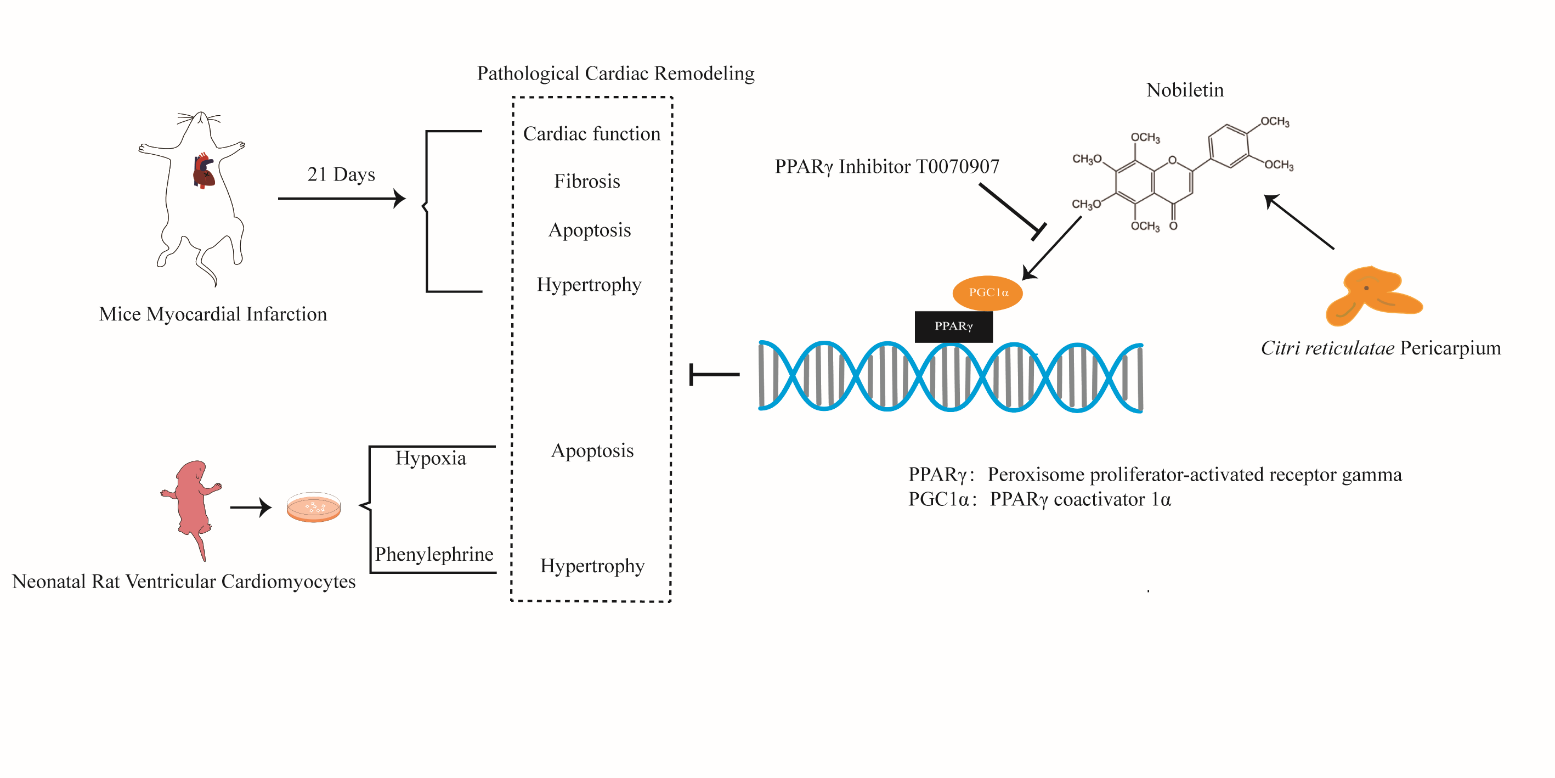


**Graphic Abstract:** Nobiletin attenuates pathological cardiac remodeling after myocardial infarction via activating PPARγ and PGC1α.
